# Case Report: Rare adverse events following CAR-T therapy in patients with relapsed/refractory diffuse large B-cell lymphoma

**DOI:** 10.3389/fonc.2025.1644818

**Published:** 2025-11-11

**Authors:** Ziniya Shah, Ranju Kunwor

**Affiliations:** 1 St Louis University School of Medicine, St. Louis, MO, United States; 2 Department of Hematology Oncology, SSM Health St Louis University Hospital, St. Louis, MO, United States

**Keywords:** DLBCL - diffuse large B cell lymphoma, CAR-T, ICANS - immune effector cell-associated neurotoxicity syndrome, immune-mediated, chimeric antigen receptor (CAR T)

## Abstract

Chimeric antigen receptor T-cell (CAR-T) therapy is a significant development in cancer therapy that is used to treat B-cell malignancies including B cell lymphoblastic leukemias, relapsed and refractory lymphomas, and multiple myeloma. CAR-T requires T-cell collection from the patient, which is genetically engineered to express a synthetic receptor that will bind to an antigen of a specific tumor. The CAR-T cells are then infused back into the patient, which function to attack the resistant cancer cells. Reported side effects of CAR-T include cytokine-release syndrome (CRS), macrophage activation syndrome (MAS), and immune effector cell-associated neurotoxicity syndrome (ICANS). Sometimes, patients can experience rare side effects which are not reported as often due to the rarity. We present a series of three patients with relapsed/refractory diffuse large B-cell lymphoma, who each received CAR-T therapy with Axicabtagene ciloleucel. Each patient developed unique and unprecedented adverse effects in the time following infusion of CAR-T. This includes manifestations of autoimmune demyelination and myelopathy, bullous dermatologic reactions on the lower extremities, and bilateral enlarged parotid glands as a result of CAR-T.

## Introduction

Diffuse large B-cell lymphoma (DLBCL) is a fast-growing hematologic malignancy that is typically treated initially with a chemoimmunotherapy regimen consisting of rituximab, cyclophosphamide, doxorubicin, vincristine, and prednisone (R-CHOP), administered over six cycles (4). Approximately 60% of patients with DLBCL achieve remission with R-CHOP. However, the remaining 40% either do not respond —10–15% are refractory—or relapse after initially achieving a complete response, accounting for 20–25% of cases (4). Chimeric antigen receptor T-cell (CAR-T) therapy has emerged as a novel immunotherapeutic in for blood cancers such as large B-cell lymphoma, mantle cell lymphoma, B-cell acute lymphoblastic leukemia, chronic lymphocytic leukemia, and multiple myeloma (1, 5).

CAR-T therapy begins with the collection of autologous T cells from the patient. The goal is to induce expression of a tumor antigen receptor in the T-cells through genetic modification. The gene encoding the desired antigen receptor is introduced into the T-cells, which are then expanded ex vivo to generate memory and effector lymphocytes that are re-infused into the patient (2). The cells expand *in vivo* and exhibit cytotoxic activity against tumor cells expressing the target antigen (6). Despite its efficacy, adverse side-effects such as cytokine release syndrome (CRS), macrophage activation syndrome (MAS), and immune effector cell-associated neurotoxicity syndrome (ICANS) have been reported. These are often due to extreme cytokine production and extensive T-cell proliferation (3). CRS commonly can present with fatigue, myalgia, headache, hypotension, high fevers, and multi-organ toxicities (7). ICANS may manifest as confusion, attention deficits, word-finding difficulties, focal deficits, encephalopathy, cerebral edema, coma, and seizures (3). ICANS grading uses the immune effector cell encephalopathy (ICE) score, assessing speech, aphasia, attention, handwriting, and orientation (8). A study showed 13% and 28% of patients who received Axi-cel for DLBCL experienced Grade 3 or higher CRS and neurologic events, respectively (9).

While common toxicities of CAR-T therapy are well-documented, less frequent or atypical adverse effects remain underreported. This case series examines rare complications following CAR-T therapy in patients with relapsed or refractory DLBCL.

## Case 1

A 51-year-old female was diagnosed with follicular lymphoma grade 1–2 in October 2023, following a CT-guided retroperitoneal mass biopsy. A PET scan showed infradiaphragmatic and supradiaphragmatic lymphadenopathy, left-sided cervical, left supraclavicular, mediastinal, retroperitoneal lymphadenopathy. The patient began treatment with bendamustine + rituxan (BR) for 5 cycles. Six months later, a CT scan showed worsening retroperitoneal lymphadenopathy (6.7 x 3.5cm), so cycle 6 was held. The patient began rituximab plus cyclophosphamide, doxorubicin, vincristine, and prednisone (R-CHOP). A para-aortic LN biopsy revealed transformation to diffuse large B-cell lymphoma (DLBCL) with CD10 +, ki-67 proliferation index >90%, C-MYC >50%, FISH showing BCL2+, BCL6+, and MYC +. Due to MYC+, R-CHOP was intensified to dose-adjusted etoposide, prednisone, vincristine, cyclophosphamide, doxorubicin, and rituximab (DA-EPOCH-R). PET/CT after C1 showed no response, so C2 was cancelled, and CAR-T therapy was considered.

**Figure 1:**
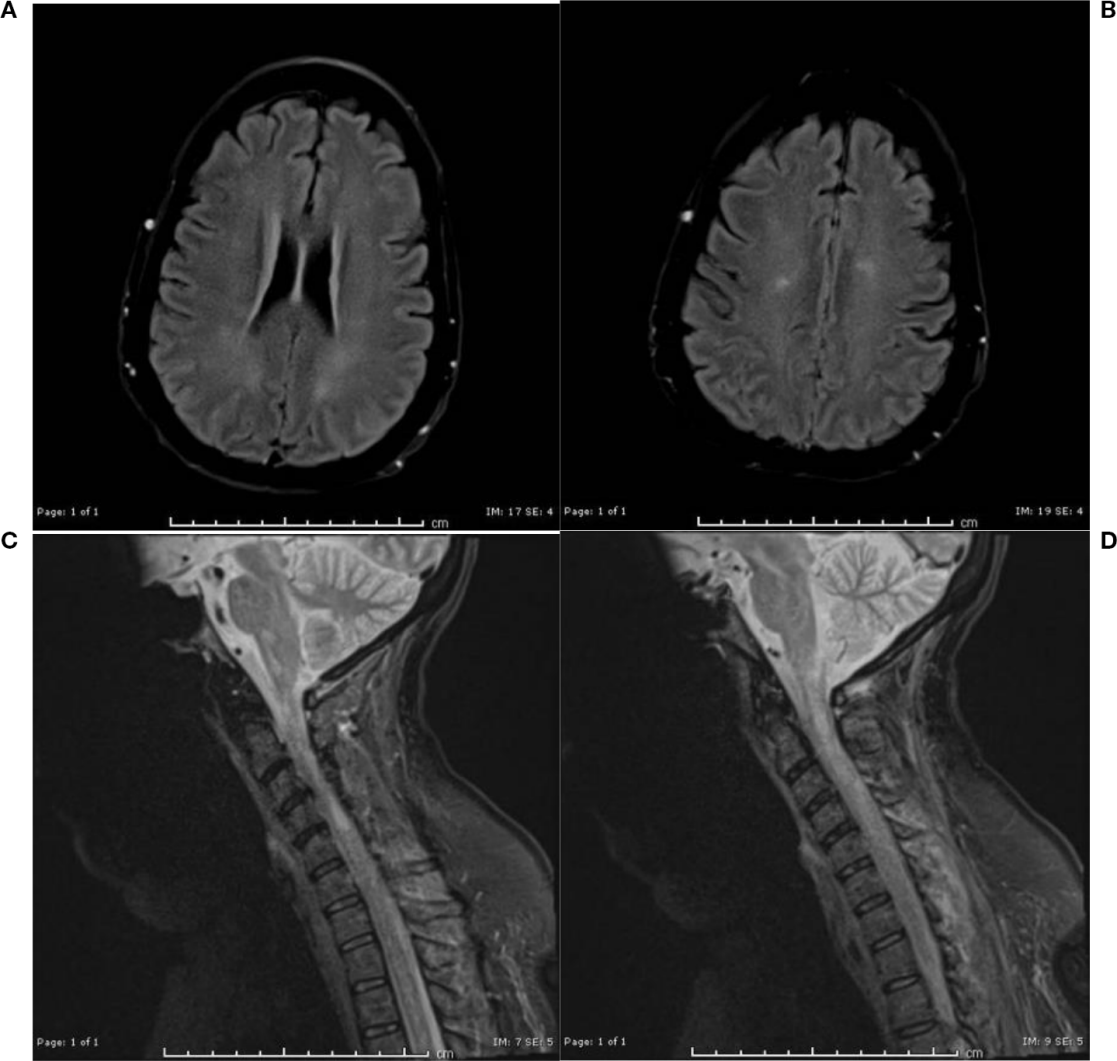
**(a, b)** MRI brain without contrast day 14+: Redemonstration of multiple foci of restricted diffusion in the lower medulla oblongata and the cervicomedullary junction. **(c, d)** MRI Cervical spine without contrast day 14: more conspicuous foci of T2/STIR hyperintensities scattered in the cervical cord, predominantly along the lateral columns of the cervical and upper thoracic cord, with corresponding associated restricted diffusion.

In June 2024, the patient received one cycle of polatuzumab vedotin 1.8 mg/kg as a bridge to CAR-T. CT showed para-aortic lymphadenopathy (7.2 cm). After T-cell collection, the patient received 3000 cGY in 10 fractions to the para-aortic nodes. Pre-CAR-T MRI brain was normal. On day -5 (5 days before CAR-T cell infusion), lymphodepleting chemotherapy was started. In September 2024, the patient received CAR-T with Axicabtagene ciloleucel. On day 0, ICE score was 10 and Keppra 750 mg BID was started as seizure prophylaxis. On day +1, she developed grade 1 CRS, treated with a dose of Tociluzumab for persistent grade I CRS. She required a second dose of tocilizumab on day +4 for persistent CRS. By day +5, her mental status declined (ICE score 1), prompting initiation of steroids (10mg of IV dexamethasone Q 6 hr, later escalated to IV methylprednisolone 1g daily). Given steroid-refractory grade 3 ICANS, Anakinra was also started (50 mg every 6 hours from days +5–10, increased to 300 mg TID on days +10–12, tapered to 200 mg BID on day +13, and 100 mg BID on days +14–15). On day +7, she received two additional doses of tocilizumab (a total of four doses; 680mg in IV 0.9% NaCl) for concurrent CRS with neurotoxicity. The patient developed myopathy and flaccidity of extremities on day +9 (ICANS grade 2). On day +10, the patient had acute onset asymmetrical pupils, and MRI of the brain revealed multiple medullary diffusion restrictions. At that time, the patient developed paraplegia. A lumbar puncture (LP) on day +12 demonstrated elevated protein and methylprednisolone was switched back to IV dexamethasone 10 mg every 6 hours An MRI on day +13 showed encephalopathy and possible immune-mediated demyelination (ICANS grade 3). Repeat MRI on day 14+ showed worsening edema and inflammation (**Figures 1a–d**), suggesting autoimmune demyelination. Despite steroids and anakinra, the patient’s symptoms persisted. A repeat LP was performed on day +15 for a more thorough workup; CSF flow and cytology was negative for malignant cells, all infectious workup in CSF was negative including adenovirus, HSV, culture/gram stain, and bacterial meningoencephalitis panel. On Day +15, methylprednisolone was restarted, and then transitioned to dexamethasone on day +17, which was then tapered to prednisone 5mg QD on day +19.

**Figure 2:**
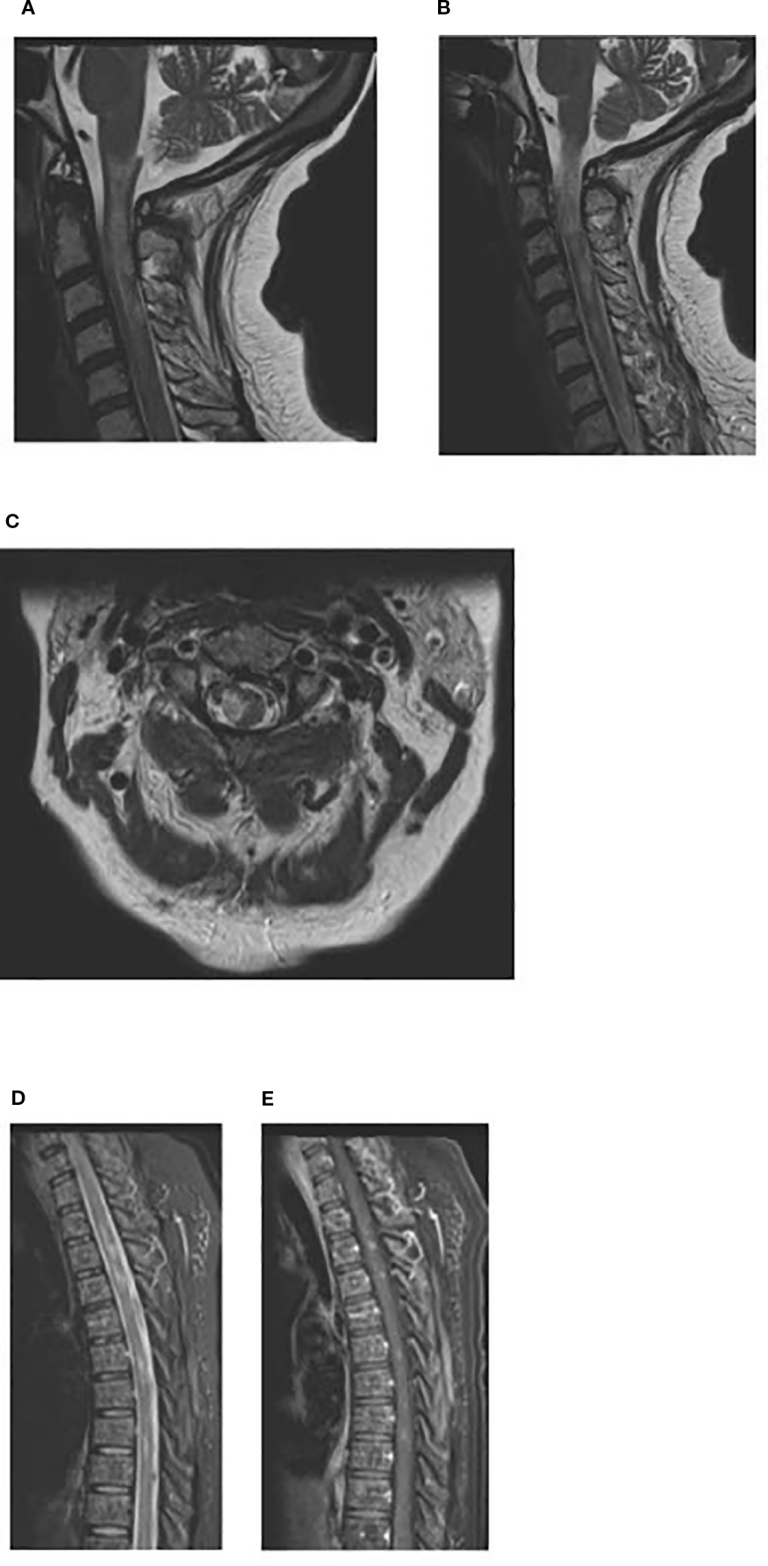
**(a, b)** MRI cervical spine day +23: Sagittal T2-weighted image of the cervical spine showing T2 hyperintense lesions in the spinal cord extending from craniocervical junction to upper cervical spinal cord suggestive of demyelinating lesions. **(c)** MRI cervical spine day +23: Axial T2-weighted image of the cervical spine at the level of C2 showing T2 hyperintense lesion in right lateral aspect/periphery of the cord which can be seen with demyelinating disease. **(d, e)** MRI thoracic spine day +23: Sagittal T2-weighted STIR image showing scattered hyperintense lesions in the spinal cord, with some of these lesions (2nd image, post contrast T1+C) showing enhancement.

An MRI on day +23 showed worsening edema (**Figures 2a–e**). Plasmapheresis (PLEX) was performed for this patient for 5 total sessions given the MRI findings and a Foley catheter was placed for urinary retention.

On day +34, the patient was discharged to rehab with oral tablets of prednisone 5 mg q.a.m. and 2.5 mg q.h.s, baclofen 5mg TID, and Keppra 750 mg BID. In follow-up appointments, she slowly regained limited extremity function. On day +40, the patient was noted to have acute myelitis along with oropharyngeal dysphagia and shortness of breath. For acute myelitis, she received IVIG over two days. She had limited movement of upper extremities (UE) and no movement of lower extremities (LE). PET scan was done on day +67 that showed complete remission (CR) of lymphoma. On day +85, the patient reported regaining some movement of her UE, but still had no movement in her LE. She remained on the prednisone 5 mg q.a.m. and 2.5 mg q.h.s, baclofen 5mg TID, and Keppra 750mg BID. By day +122, she had improved movement in her left hand and some in her right. She was able to move her toes, but frequently experienced spasms in her legs and feet. By day +154, she could move bilateral toes, had near full left UE function, but no leg movement. On day +184, she had 1/5 strength in right UE and LE, and bladder incontinence. A PET scan on day +154 showed ongoing CR. On day +210, the patient’s labs and assessment suggested ongoing remission for the DLBCL. By day +210, she remained in remission with continued neurologic recovery, showing near full left UE and increasing right UE and left LE movement.

## Case 2

A 63 year-old male presented with an axillary lump in July 2023. CT scan showed extensive lymphadenopathy above and below the diaphragm and splenomegaly. Lymph node biopsy identified DLBCL CD45+, CD20+, BCL2+, ki-67 proliferation index ~ 40%. A PET scan prior to treatment showed splenic enlargement and increased FDG uptake in multiple lymph nodes throughout the neck, chest, abdomen, and pelvis, consistent with DLBCL stage IV. The patient received 6 cycles of R-CHOP. A follow-up PET scan showed markedly decreased adenopathy with mild residual FDG activity (right external iliac lymph node measured 5 mm, SUV max 2.7; prior SUV max was 8.9), consistent with response to treatment. A repeat PET scan two months later showed disease progression with increased FDG uptake in the mediastinum, neck, and left axilla, and a new FDG-avid node in the right external iliac chain. CAR-T was then discussed for DLBCL refractory to R-CHOP.

**Figure 3:**
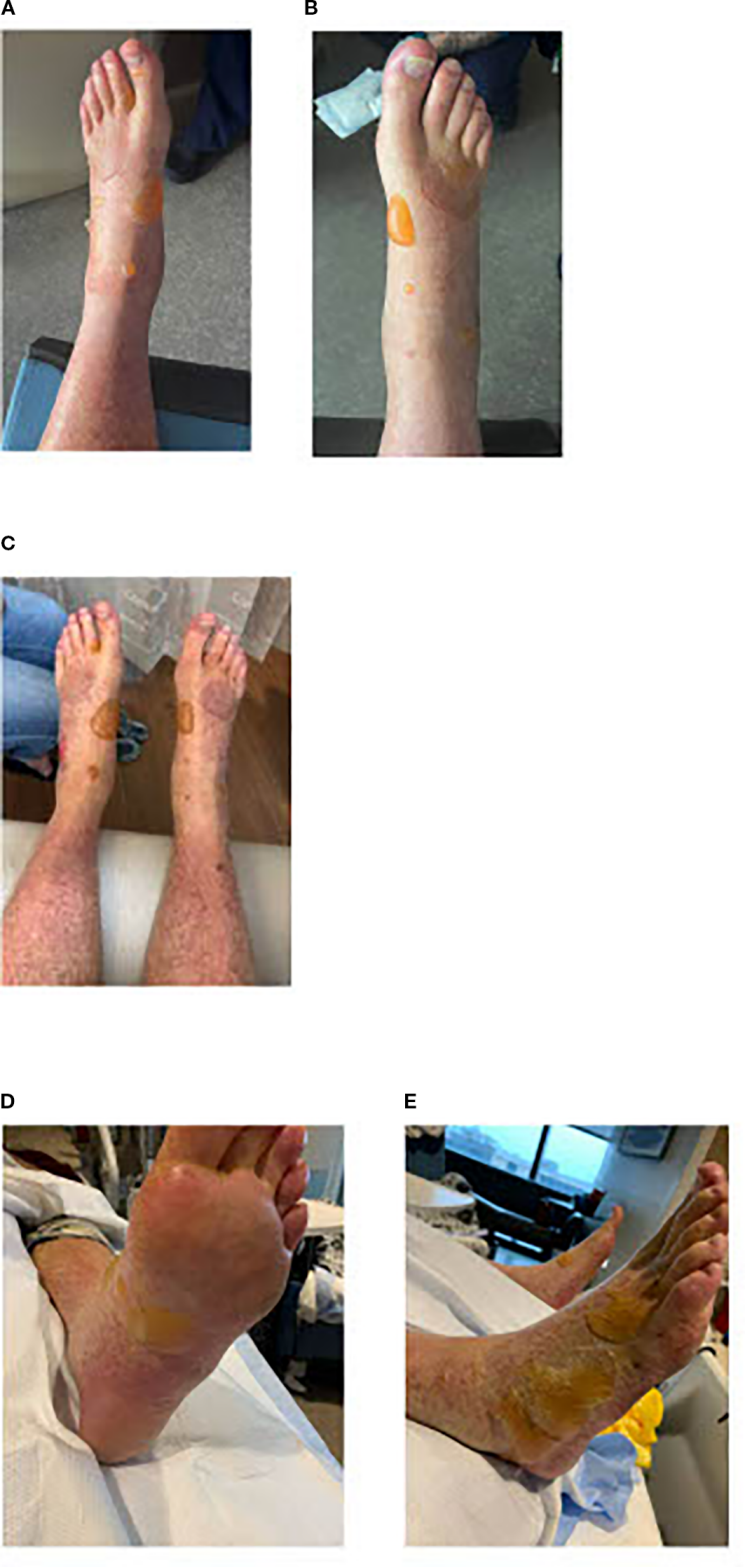
**(a, b)** Day +10: Fluid-filled bullae on bilateral lower extremities. **(c)** Day +15: Blisters on bilateral lower extremities. **(d, e)** Day +21: Bilateral lower extremities blisters that are showing some improvement.

The patient underwent T-cell collection for CAR-T. A PET scan showed further disease progression, prompting one cycle of rituximab, ifosfamide, carboplatin, and etoposide (R-ICE) chemotherapy prior to CAR-T. A follow-up PET scan showed interval decrease in lymphadenopathy above and below the diaphragm, consistent with partial response and a Deauville score of 4. In mid-September 2024, the patient received CAR-T with Axicabtagene ciloleucel. The patient developed CRS on day +1, and was treated with Tociluzumab (800mg in IV 0.9 NaCl; dose 1 on day +1, dose 2 on day +3, dose 3 on day +4, dose 4 on day +6). On day +5, the patient showed signs of neurotoxicity with manifestations of illegible sentences and mental status decline; IV dexamethasone 10mg Q6hr was started. The patient also developed elevated liver enzymes, hyperbilirubinemia, and fluid overload. By day +8, his mental status improved. On day +10, the patient developed large, fluid-filled bullae on both lower extremities (**Figures 3a, b**), causing difficulty with ambulation and pain. The blisters had no sign of infections (as seen in the figures). Biopsies were not performed due to the patient being treated for neurotoxicity. Because these were not rapidly spreading, skin lesions were only watchfully observed.

The patient was discharged on day +14 and steroids were tapered off at discharge. At a follow-up on day +15, the patient reported continued difficulty with walking due to the fluid-filled blisters and several popped ones that were causing redness and peeling of the skin (**Figure 3c**).

On day +21, the blisters were less painful and the physical exam showed improvement (**Figures 3d, e**). In follow-ups, the patient reported continued improvement in strength and foot pain. A PET scan on day +30 showed no evidence of disease, consistent with complete remission. On day +31, the skin lesions were fully resolved. A repeat PET scan on day +100 confirmed continued complete remission.

## Case 3

A 62-year-old man presented with abdominal pain, with CT imaging finding of adenopathy in his abdomen and pelvis. An ultrasound-guided biopsy of intra-abdominal lymph nodes showed diffuse large B-cell lymphoma stage IIIb, that was CD20 +, Bcl-2 + and Mum-1 + by IHC. He completed 6 cycles of R-CHOP. A PET scan post R-CHOP showed that hypermetabolic retroperitoneal and mesenteric adenopathy was completely resolved, with no evidence of active lymphoma in the abdomen or pelvis. However, there were some mildly hypermetabolic hilar lymph nodes, with a slightly higher uptake than the liver. This remained stable in subsequent surveillance scans. A later PET scan revealed a right flank mass, consistent with a cluster of lymph nodes. A peritoneal biopsy confirmed that the lymphoma had relapsed, showing recurrent diffuse large B-cell lymphoma.

**Figure 4:**
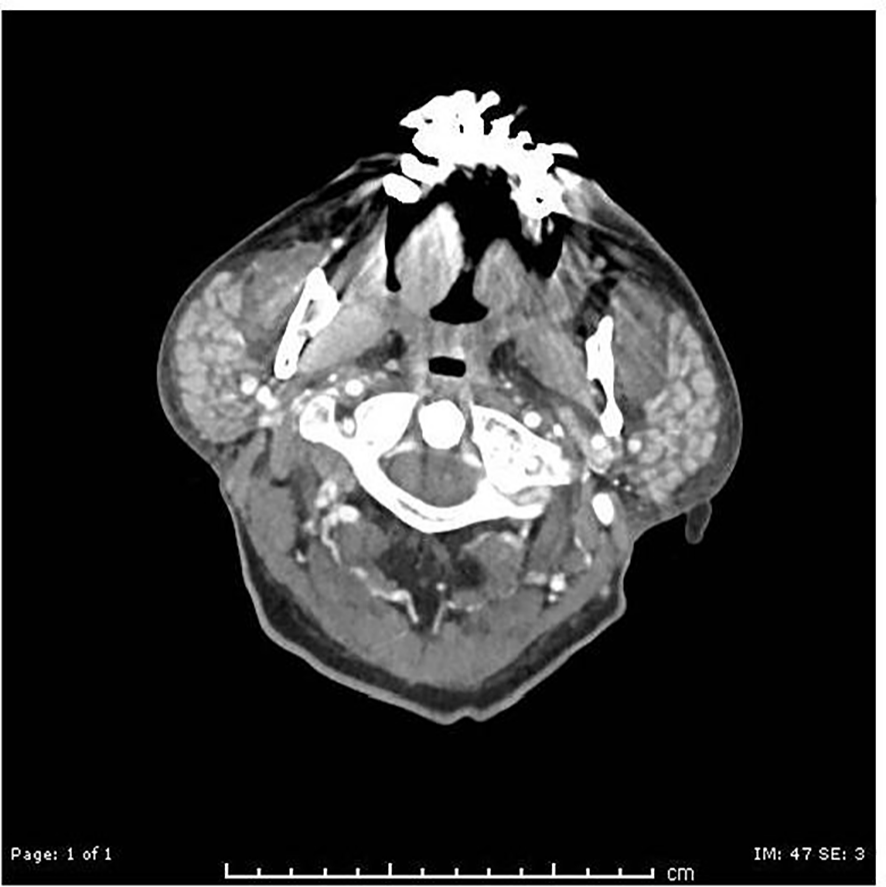
CT day +4: symmetric enlargement of the bilateral parotid glands, with numerous small hyperdense nodules.

The patient underwent CAR-T cell collection and around a month later, the patient began lymphodepleting chemotherapy followed by CAR-T cell infusion. In mid-April 2024 (Day +0), the patient received CAR-T Axicabtagene ciloleucel. On day +2, the patient was febrile, tachycardic, and hypotensive and was determined to have CRS grade 2 and received standard treatment with Tociluzumab 8mg/kg/dose (600mg in IV 0.9 NaCl; dose 1 on day +3, 2 doses (560mg and 600mg) on day +4, and 1 doses on day +14), and steroids (IV dexamethasone 10mg for 13 days). The patient had clinical bilateral parotid enlargement; a CT of the neck and face on day +4 (**Figure 4**) showed symmetric enlargement of the parotid glands, with numerous, small hyperdense nodules and no evidence of abscess. The parotid swelling was watchfully observed, while continuing treatment for CRS and neurotoxicity. On day +5, the patient had confusion and speech abnormality; EEG showed bifrontally and midline predominant epileptiform discharges, and generalized slowing. MRI on day +5 showed mild paranasal sinus disease with mucous retention cyst or polyp in the left maxillary sinus. By Day +13, ICANS and CRS improved. Parotid swelling subsided. Steroids were tapered off over the next few days. On day +17, the patient was discharged. On day +38, the patient’s PET scan showed improvement of disease, with a Deauville score of 3 consistent with CR, and completely normal parotid glands. On day +364, the surveillance PET scan showed continued CR, indicating remission of DLBCL.

## Discussion

Chimeric antigen receptor T-cell (CAR-T) therapy has shown promise for treating refractory or relapsed diffuse large B-cell lymphoma cancer (DLBCL). Axicabtagene ciloleucel (Axi-cel, Yescarta) is an anti-CD-19 CAR-T therapy (10). CD-19 is an antigen found on both normal and malignant B-cells. Axi-cel binds to CD19 positive cells and its CD28 and CD3ζ costimulatory domains, which drives T-cell activation and proliferation, promoting cytokine secretion and subsequent death of CD19-positive cells (10). A study showed the outcomes of CAR-T Axi-cel through objective response rate and complete response which were 82% and 54% respectively (10). Despite its efficacy, CAR-T can be associated with significant adverse effects: cytokine release syndrome (CRS) and immune effector cell-associated neurotoxicity syndrome (ICANS). These side effects have been well-documented along with effective treatment strategies. However, the spectrum of CAR-T therapy’s adverse events is still incompletely understood, especially when it comes to rare, under-reported complications.

This case series presented three patients who had relapsed or refractory DLBCL and developed uncommon toxicities following CAR-T therapy. All side effects occurred within 30 days of receiving CAR-T therapy and are unusual events that have not been previously reported. The first case was a 51 year-old woman who experienced a severe neurotoxicity that involved progressive weakness, encephalopathy, quadriplegia, and immune-mediated demyelination. The patient was administered corticosteroids, Anakinra, and IVIG, with only partial improvement of the neurotoxicity. ICANS is a recognized complication of CAR-T therapy, and typically presents with confusion, aphasia, seizures, and edema (11). In this patient, the extent of demyelination and neuroinflammation is atypical and suggests a complex autoimmune process involving T-cells. Demyelination involves immune-mediated injury to the myelin sheath. While the pathophysiology is not entirely understood, it may involve a proinflammatory cytokine environment. CAR-T therapy is associated with higher levels than baseline of C-reactive protein, ferritin, proinflammatory cytokines (such as interferon-gamma, IL-6, IL-1, IL-10, and MCP-1), and indicators of endothelial cell activation (12–17). Elevated proinflammatory markers in CSF can be associated with blood-cerebrospinal barrier dysfunction – which is associated with neurotoxicity (16) and this disruption may be triggering autoimmune attacks. IVIG for neurotoxicity refractory to corticosteroids and Anakinra has been helpful for more extreme cases of ICANS (18), but the delayed recovery in this patient further suggests a complex autoimmune process. The second patient developed fluid-filled bullae on bilateral lower extremities after CAR-T infusion, resulting in impaired ambulation, pain, and skin breakdown. Wound care and wheelchair usage allowed the blisters on the patient’s feet to heal, but the presence is still atypical. The environment of high levels of cytokines could provide opportunity for immune-mediated bullous dermatosis. This did not require treatment and no biopsy was performed to prove the immune mediated bullae formation. Use of immunosuppressant drugs including steroids, and IL-6 inhibitors itself might have helped in treating the skin lesions and ultimately, resolved the bullae. The third case was a patient who developed bilateral parotid glands enlargement; MRI revealed nodularity and sinus abnormalities. Similar to the other cases, the pathophysiology is not yet fully understood, but the elevated cytokines as a result of T-cell activation could have resulted in immune-mediated adverse effects. This case was also watchfully observed and concurrent management of CRS/neurotoxicity sufficed in settling the parotid inflammation.

The use of corticosteroid post-CAR-T infusion may have a prognostic impact. A retrospective study observed that usage of corticosteroids within the first 30 days after CAR T-cell infusion is associated with shorter overall survival. Additionally, a higher cumulative dose and prolonged and early use of corticosteroids were associated with a shorter progression-free survival and/or overall survival (19). However, early use of corticosteroids for low-grade CRS in combination with Tocilizumab after CAR-T could reduce the risk of high-risk CRS without negatively impacting neurotoxicity or treatment outcome (20). All three of the patients had complete response post-CAR-T and remain in remission.

None of the patients received a prophylactic dose of dexamethasone or steroids. Even the prophylactic steroid use has shown to have no effect on the efficacy of CAR-T therapy (21). Although retrospective studies suggest prolonged or high-dose corticosteroids may impair outcomes, emerging evidence indicates that short-term or prophylactic steroid use does not negatively impact CAR-T efficacy. Despite steroid administration, all three patients achieved durable complete remission.

These three cases highlight the diverse spectrum of immune-mediated adverse events that may occur after CAR-T, while underscoring the need for clinical awareness beyond the well-characterized manifestations of CRS and ICANS. Notably, all 3 cases of adverse events happened within 30 days of CAR-T infusion, representing early and uncommon complications. To our knowledge, quadriparesis due to immune-mediated demyelination following CAR-T cell therapy has not been extensively reported. In addition to the more common presentations of CRS and ICANS, rare complications have been reported. Beyond typical manifestations of ICANS, rare complications described in the literature include non-convulsive status epilepticus, bilateral facial nerve palsy, diabetes insipidus, and Guillain-Barre-like syndrome, extensive myelitis with eosinophilic pleocytosis, and oropharyngeal and laryngeal dystonia (22). These additional side effects may reflect autoimmune processes triggered by T-cell activation and blood-CSF barrier disruption. The extent of quadriplegia that our patient faced is a very rare side-effect and has not been reported on prior to this. Additionally, parotitis is an uncommon side-effect and does not have much literature; it has typically been resolved with supportive care or steroids (23). Dermatologic manifestations, including bullous skin reactions and even toxic epidermal necrolysis (TEN) have been reported post CAR-T (24–26). This suggests immune-mediated skin injury in the context of cytokine elevation. Our cases expand this spectrum, illustrating severe quadriplegia, parotitis, and bullous dermatosis as early-onset, immune-mediated complications. These reports contextualize our cases and emphasize the need for early detection and intervention for atypical CAR-T related toxicities.

In the cases that we presented, it is possible that the immune activation triggered by CAR-T therapy extends beyond intended cytotoxic effects, expanding to immune-mediated syndromes. As CAR-T therapy is relatively new, previously unreported side effects may emerge. These cases highlight the severity of rare side-effects of CAR-T related therapies. This includes, but is not limited to, autoimmune neurologic conditions, atypical inflammatory processes, and bullous dermatologic reactions. To ensure preparation for potential side effects and optimization of recovery, clinicians should be aware of the unique side-effects that uncommonly occur due to CAR-T. As the popularity of CAR-T continues to increase, further research is warranted on the therapy-related toxicity to refine care strategies following unanticipated side-effects.

## Data Availability

The original contributions presented in the study are included in the article/supplementary material. Further inquiries can be directed to the corresponding author.
